# Insulin sensitivity, disposition index and insulin clearance in cystic fibrosis: a cross-sectional study

**DOI:** 10.1007/s00125-024-06220-6

**Published:** 2024-08-02

**Authors:** Bibi U. Nielsen, Inger H. M. Mathiesen, Rikke Krogh-Madsen, Terese L. Katzenstein, Tacjana Pressler, James A. M. Shaw, Michael R. Rickels, Thomas P. Almdal, Daniel Faurholt-Jepsen, Darko Stefanovski

**Affiliations:** 1 Cystic Fibrosis Centre Copenhagen, Department of Infectious Diseases, Copenhagen University Hospital – Rigshospitalet, Copenhagen, Denmark; 2 Centre for Physical Activity Research, Copenhagen University Hospital – Rigshospitalet, Copenhagen, Denmark; 3 Department of Infectious Diseases, Copenhagen University Hospital – Hvidovre, Copenhagen, Denmark; 4 Translational and Clinical Research Institute, Newcastle University, Newcastle upon Tyne, UK; 5 Division of Endocrinology, Diabetes & Metabolism, Department of Medicine, and Institute for Diabetes, Obesity & Metabolism, University of Pennsylvania Perelman School of Medicine, Philadelphia, PA USA; 6 Department of Endocrinology, Copenhagen University Hospital – Rigshospitalet, Copenhagen, Denmark; 7 Department of Clinical Studies-New Bolton Center, University of Pennsylvania School of Veterinary Medicine, Kennett Square, PA USA

**Keywords:** CFRD, Disposition index, Insulin clearance, Insulin secretion, Insulin sensitivity, Minimal models

## Abstract

**Aims/hypothesis:**

The aim of this study was to investigate insulin secretion, insulin sensitivity, disposition index and insulin clearance by glucose tolerance status in individuals with cystic fibrosis (CF) and exocrine pancreatic insufficiency.

**Methods:**

In a cross-sectional study, we conducted an extended (ten samples) OGTT in individuals with pancreatic-insufficient CF (PI-CF). Participants were divided into normal glucose tolerance (NGT), early glucose intolerance (EGI), impaired glucose tolerance (IGT) and CF-related diabetes (CFRD) groups. We used three different oral minimal models to assess insulin secretion, insulin sensitivity and insulin clearance during the OGTT. We evaluated insulin secretion using total secretion (Φ total), first-phase secretion (Φ dynamic) and second-phase secretion (Φ static) from the model, and we estimated the disposition index by multiplying Φ total and insulin sensitivity.

**Results:**

Among 61 participants (NGT 21%, EGI 33%, IGT 16%, CFRD 30%), insulin secretion indices (Φ total, dynamic and static) were significantly lower in the CFRD group compared with the other groups. Insulin sensitivity declined with worsening in glucose tolerance (*p* value for trend <0.001) and the disposition index declined between NGT and EGI and between IGT and CFRD. Those with CFRD had elevated insulin clearance compared with NGT (*p*=0.019) and low insulin secretion (Φ total) was also associated with high insulin clearance (*p*<0.001).

**Conclusions/interpretation:**

In individuals with PI-CF, disposition index declined with incremental impairment in glucose tolerance due to a reduction in both insulin secretion and insulin sensitivity. Moreover in CF, reduced insulin secretion was associated with higher insulin clearance.

**Graphical Abstract:**

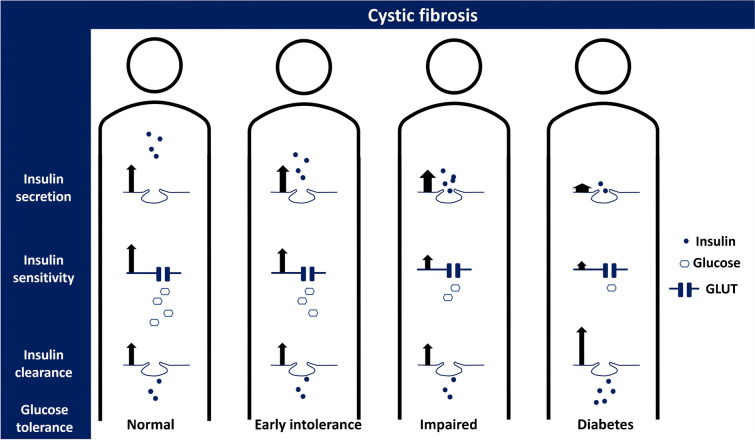

**Supplementary Information:**

The online version of this article (10.1007/s00125-024-06220-6) contains peer-reviewed but unedited supplementary material.


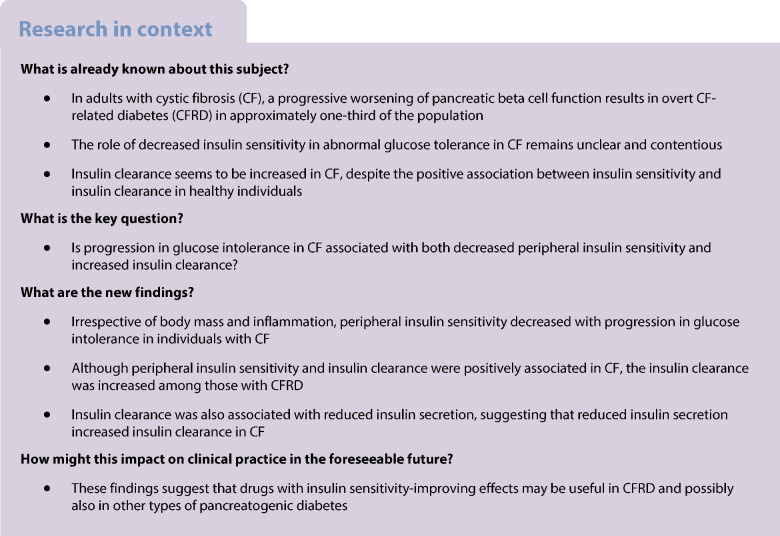


## Introduction

A common comorbidity in cystic fibrosis (CF) is CF-related diabetes (CFRD) [1], which aggravates abnormalities in nutritional status and lung capacity [2]. Insufficient insulin secretion seems to cause the progression to CFRD [3, 4] but changes in insulin sensitivity and insulin metabolism kinetics may also contribute to pathogenesis [5]. To improve understanding of CFRD, a more profound insight into insulin metabolism in CF is needed.

Previous CF studies investigating the association between impaired glucose tolerance (IGT) and insulin sensitivity have been divergent. Several of these studies indicated that insulin sensitivity decreases with glucose intolerance, as IGT and CFRD were associated with lower peripheral insulin sensitivity compared with normal glucose tolerance (NGT) in euglycaemic clamp studies [6, 7]. This said, a more recent study with hyperglycaemic clamps did not find a difference in insulin sensitivity across groups with worsening glucose tolerance in CF [4]. In addition, prospective studies by Boudreau et al [8] and Colomba et al [9] showed that worsening glucose tolerance was related to a decrease in insulin sensitivity rather than a decline in insulin secretion. On the other hand, studies using simple indices derived from OGTTs have shown unaltered fasting and non-fasting insulin sensitivity across glucose tolerance groups [10–12]. Thus, it is still not clear whether altered insulin sensitivity impairs glucose tolerance in CF [13]. We hypothesised that insulin sensitivity and the disposition index, which is the product of insulin secretion and insulin sensitivity, worsen with progression in glucose intolerance in CF.

Few studies have examined the kinetics of insulin metabolism in CF [14]. Dysregulated glucose metabolism may affect both the insulin clearance and the hepatic extraction fraction in CF, similar to what is seen in non-CF populations [15, 16]. Low insulin levels seem to increase insulin clearance in healthy individuals [17], suggesting that insufficient insulin secretion may lead to an increase in insulin clearance in individuals with CF. Consistent with this, a CF study showed that the whole-body (i.e. liver, kidney and skeletal muscle) insulin clearance was elevated in individuals with CF compared with healthy control individuals [18, 19]. However, it is not known whether insulin clearance and hepatic extraction fraction change with progressive impairment in glucose tolerance in CF.

With mathematical modelling, we assessed total, dynamic and static insulin secretion as well as insulin sensitivity, disposition index, insulin clearance and hepatic extraction fraction in different glucose tolerance groups of individuals with PI-CF. Furthermore, we assessed the associations between insulin secretion and insulin kinetic variables (insulin clearance and hepatic extraction fraction) in CF.

## Methods

### Participants

In a cross-sectional study at the Copenhagen CF Center, we invited adults (aged >18 years) with genetically confirmed CF to participate in an extended OGTT. The exclusion criteria were current pregnancy, solid organ transplantation and pancreatic sufficiency (defined as faecal elastase >200 μg/g in the most recent sample). To maximise the power of the study, we included all eligible and consenting individuals irrespective of sex, and hence no power calculation was done in advance.

### Data collection

We obtained participant characteristics from the Danish CF Registry, including age, sex, mutation class, faecal elastase (ScheBo ELISA; ScheBo Biotech, Germany), BMI, alkaline phosphatase (Cobas 8000, c702 module; Roche Diagnostics, Switzerland), γ-glutamyl transferase (GGT) (Cobas 8000, c502 module; Roche Diagnostics), eGFR according to the CKD-EPI equation based on plasma creatinine (Cobas 8000; Roche Diagnostics) and lung function assessed with spirometry (Intramedic Vyntus SPIRO, Standard EU‐GLI; Vyaire Medical, Germany).

An extended OGTT was performed to assess glucose tolerance, insulin secretion, insulin sensitivity and insulin metabolism. Prior to the OGTT, participants were fasted overnight and long-acting insulin was withheld for 24 h and short-acting insulin for 12 h. First, two fasting blood samples were collected (−10 min and −1 min) before the ingestion of 75 g glucose diluted in water over a 2 min period. This was followed by blood sampling at 10, 20, 30, 45, 60, 90, 120, 150 and 180 min after ingestion. Samples were placed on ice immediately after collection and centrifuged within 1 h. Thereafter, the samples were frozen at −80°C until further analysis. We measured glucose (e702 module) and C-peptide (e801 module) in serum and insulin (e801 module) in plasma using Cobas 8000 (Core Chemistry Analyzer; Roche Diagnostics).

We defined glucose tolerance status using the following criteria: NGT, 1 h glucose <8.6 mmol/l and 2 h glucose <7.8 mmol/l; early glucose intolerance (EGI), 1 h glucose ≥8.6 mmol/l and 2 h glucose <7.8 mmol/l; IGT, 2 h glucose 7.8–11.0 mmol/l; and CFRD, 2 h glucose ≥11.1 mmol/l [3]. Instead of indeterminate glucose intolerance, we used EGI, as this group with a near-normal glucose tolerance already has established beta cell secretory defects in comparison with NGT [4]. HOMA-IR, the Matsuda index and the Stumvoll index for insulin sensitivity [20] were calculated as described in the ESM Methods.

Lastly, a traditional and simple estimate of the insulin clearance was calculated with:

$$\frac{\text{Total AUC}_{\text{C-peptide}}}{\text{Total AUC}_{\text{Insulin}}}$$

These simple indices of insulin resistance, insulin sensitivity and insulin clearance were calculated to evaluate the correlation between simple indices and estimates from mathematical models.

### Mathematical models

Three different mathematical models were fitted with WinSAAM version 3.3.0 (University of Pennsylvania, USA). Insulin secretion was estimated in Breda’s model using C-peptide and glucose concentrations [21]. Breda’s model splits insulin secretion into an estimate of the first-phase secretion (Φ dynamic) and the second-phase secretion (Φ static), with Φ total representing the combination of both. Insulin sensitivity was estimated in the Dalla Man’s model using insulin and glucose concentrations, assuming 90% glucose absorption [22], and the disposition index was defined as the product of Φ total and insulin sensitivity. Insulin clearance and hepatic extraction fraction were estimated with Watanabe’s model using insulin and C-peptide concentrations [23]. An advantage of this model is the estimate of hepatic extraction, which is not obtained from the simple insulin clearance AUC ratio equation. Breda’s and Watanabe’s models assume that the C-peptide kinetics follow a two-compartment distribution and Watanabe’s model assumes that insulin has a linear single-compartment elimination. The parameters from the mathematical models were used as the primary outcomes in the study.

### Statistical analyses

The statistical analyses were calculated in R (version 4.2.2, available at https://www.r-project.org/). Normality of data was checked with quantile–quantile (Q-Q) plots. Participant characteristics were described with proportions or means and geometric means ± SD as appropriate. Differences in participant characteristics by glucose tolerance group were tested in logistic or linear regression models (positively skewed data was transformed) using the glucose tolerance group as an ordinal exposure variable. In addition, time to peak (T_Max_) and maximum values of the dynamic and static insulin secretion rate (ISR_Max_) were estimated. As Φ dynamic and insulin sensitivity followed a positively skewed distribution, they were log-transformed and the back transformed estimates were presented for these variables. All mathematical model parameters were summarised in linear models with the glucose tolerance group as the independent variable and the parameter as the outcome using robust SEs. The marginal means of the models were calculated and shown in bar charts. The disposition index was estimated by fitting the reciprocal function: Φ total = β/Insulin sensitivity with the vertical and horizontal asymptotes being equal to zero. The associations between metabolic variables (insulin secretion, insulin sensitivity, disposition index) as the fixed effects and insulin clearance/hepatic extraction fraction status as the outcomes were fitted in linear models. The linear models were adjusted for BMI using robust SEs. The associations were further confounded for age, sex and insulin sensitivity in multivariable analyses. In a sensitivity model, we estimated insulin clearance with a fixed hepatic extraction of 50% [24] and insulin sensitivity indices (i.e. HOMA-IR, Matsuda index, Stumvoll index) were compared with the modelled insulin sensitivity with Spearman’s correlation. We also compared insulin clearance and the hepatic extraction fraction with Spearman’s correlation and examined these in the CFRD subgroups stratified by insulin treatment dependency. Differences in metabolic variables between glucose tolerance groups were calculated for all possible combinations but only significant *p* values were reported in the figures. Only *p* values <0.05 (two-tailed) were considered statistically significant.

### Ethical considerations

Ethical approval (H-19085530) was provided by the ethical committee in the Capital Region of Denmark. Only participants who gave written informed consent after oral and written information were included in the study.

## Results

**Fig. 1:**
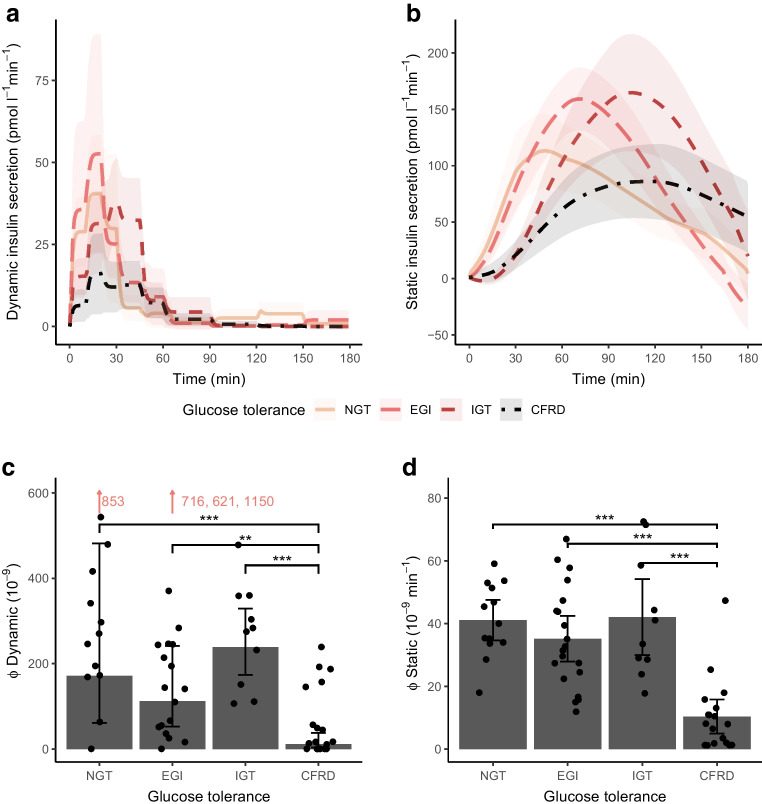
First-phase (**a**, **c**) and second-phase (**b**, **d**) insulin secretion by glucose tolerance status during an extended OGTT in 61 individuals with PI-CF. Data are presented as means (95% CI) calculated with unadjusted linear regression models using robust SE. Φ dynamic was log-transformed and back transformed in the model. Outliers are indicated by red numbers. **p<0.01, ***p<0.001

Between August 2020 and January 2021, 61 individuals with PI-CF underwent an extended OGTT (flow chart is shown in electronic supplementary material [ESM] Fig. 1). Compared with the entire Danish CF population, the average age of the study population was higher, and the proportion of women were lower in the EGI, IGT and CFRD group. We did not have information on residential region and socioeconomic status. Ninety-seven per cent of the participants originated from Europe and the remaining participants originated from the Middle East. Thirteen (21%) were categorised as NGT, 20 (33%) as EGI, 10 (16%) as IGT and 18 (30%) as CFRD. The majority had normal BMI (median 22.3 kg/m^2^ [IQR 20.8–24.1 kg/m^2^]) and CF liver disease biomarkers were generally normal, with the upper quartile of alkaline phosphatase and GGT being within normal range. HbA_1c_ increased with glucose intolerance (*p*<0.001) but inflammation status, BMI and lipid profiles were similar between groups (Table 1).

**Table 1:** Characteristics of 61 individuals with CF and exocrine pancreatic insufficiency

Characteristic	NGT (*n*=13)	EGI (*n*=20)	IGT (*n*=10)	CFRD (*n*=18)	*p* value for trend
Demographics					
Age, years	28 ± 7	28 ± 8	43 ± 14	35 ± 9	<0.001
Female sex, *n* (%)	7 (54)	7 (35)	3 (30)	4 (22)	0.088
Mutation, *n* (%)					
Homozygous F508del	11 (85)	11 (55)	8 (80)	13 (72)	0.727
Heterozygous F508del	1 (8)	8 (40)	2 (20)	5 (28)	0.335
Lung function					
FEV_1_%	93 ± 26	73 ± 23	69 ± 25	75 ± 21	0.050
Inflammation status					
C-reactive protein, mg/l	1.6 ± 1.9	2.3 ± 2.8	5.6 ± 2.6	2.2 ± 2.8	0.069
Leucocytes, 10^9^/l	6.9 ± 1.3	6.2 ± 1.3	7.5 ± 1.4	7.0 ± 1.5	0.507
Diabetes status					
HbA_1c_, mmol/mol	37.1 ± 1.1	37.8 ± 1.1	39.5 ± 1.1	50.4 ± 1.3	<0.001
Fasting glucose, mmol/l	5.1 ± 1.1	5.4 ± 1.1	5.6 ± 1.1	7.7 ± 1.3	<0.001
Insulin treatment^a^					
Insulin treated, *n* (%)	0 (0.0)	0 (0.0)	0 (0.0)	11 (61.1)	NA
Insulin treatment period, years	NA	NA	NA	10.0 (6.0-20.0)	NA
Short-acting insulin dose, U/day	NA	NA	NA	16.5 (10.5-23.2)	NA
Long-acting insulin dose, U/day	NA	NA	NA	11.0 (10.0-25.2)	NA
Nutritional status					
BMI, kg/m^2^	22.7 ± 3.2	22.4 ± 2.7	22.9 ± 2.9	22.4 ± 2.7	0.880
Lipid profile					
Total cholesterol, mmol/l^b^	3.3 ± .7	3.3 ± .8	3.8 ± 1.0	3.5 ± .6	0.221
LDL-cholesterol, mmol/l^b^	1.7 ± .6	1.7 ± .7	2.1 ± .9	1.8 ± .3	0.280
Organ function					
Alkaline phosphatase, U/l^b^	94 ± 1	101 ± 2	114 ± 2	135 ± 2	0.021
GGT, U/l^b^	19 ± 2	25 ± 3	28 ± 2	45 ± 4	0.019
eGFR, ml/min per 1.73 m^2b^	87 ± 10	87 ± 6	81 ± 12	80 ± 18	0.099
Indices					
HOMA-IR	1.4 ± 1.8	1.2 ± 1.7	1.2 ± 1.9	1.1 ± 2.4	0.327
Matsuda index	9.0 ± 1.6	7.2 ± 1.6	7.8 ± 1.4	10.9 ± 1.9	0.319
Stumvoll index	0.10 ± 0.02	0.10 ± 0.02	0.08 ± 0.01	0.04 ± 0.03	<0.001

Data are shown as means or geometric means ± SD, as appropriate, or as *n* (%) Insulin treatment period and doses were reported with median (IQR)

Data were tested in linear or logistic regression models with glucose tolerance as an ordinal exposure

^a^Among those who were treated with insulin, seven were treated with insulin degludec and three were treated with insulin detemir

^b^Most recent assessment prior to the OGTT

FEV_1_%, forced expiratory volume in 1 s

**Fig. 2:**
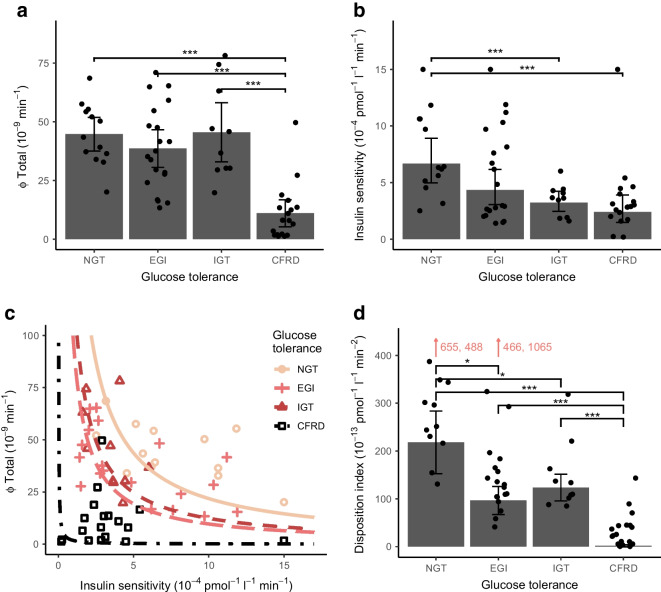
Total insulin secretion (**a**), insulin sensitivity (**b**), disposition index curves (**c**) and disposition index (**d**) by glucose tolerance status during an extended OGTT in 61 individuals with PI-CF. Data are presented as means (95% CI) calculated with unadjusted linear regression models using robust SE. Insulin sensitivity was log-transformed and back transformed in the model. The disposition index was calculated with an inverse regression with no constant. Outliers are indicated by red numbers. *p<0.05, ***p<0.001

Glucose, insulin and C-peptide concentrations are shown in ESM Fig. 2. The dynamic and static insulin secretion rate and the values of Φ dynamic and Φ static are shown by glucose tolerance status in Fig. 1. Φ dynamic, Φ static and Φ total were unchanged among those with NGT, EGI and IGT, while they were all reduced in those with CFRD compared with the other groups (Figs 1, 2a). Moreover, the static secretion peaked later with increasing glucose intolerance (*p* value for trend <0.001) (ESM Fig. 3).

**Fig. 3:**
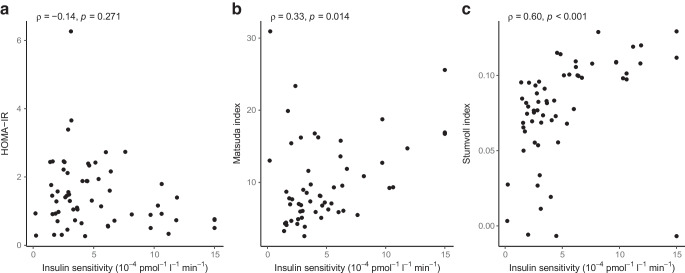
Spearman’s correlation between modelled insulin sensitivity and HOMA-IR (**a**), Matsuda index (**b**) and Stumvoll index (**c**) in 61 individuals with PI-CF. Matsuda index was based on samples at −1, 30, 60, 90 and 120 min

Insulin sensitivity declined with glucose intolerance (*p* value for trend <0.001) and was significantly lower in the IGT and CFRD groups compared with the NGT group (Fig. 2b). The disposition index (hyperbola coefficient) was lower in the EGI group than in the NGT group; it was also lower in the CFRD group than in the IGT group but was unchanged between the EGI and IGT groups (Fig. 2c, d). Neither the HOMA-IR nor the Matsuda index was associated with glucose tolerance status (*p* value for trend=0.327 and 0.319, respectively). However, the Stumvoll index decreased with glucose intolerance (*p* value for trend *p*<0.001). The modelled insulin sensitivity correlated with the Matsuda index (*R*=0.33, *p*=0.014) and Stumvoll index (*R*=0.6, *p*<0.001) but not with HOMA-IR (*R*=−0.14, *p*=0.271) (Fig. 3).

**Fig. 4:**
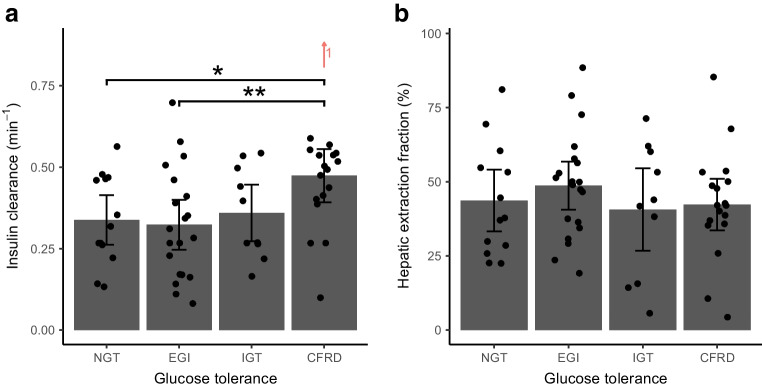
Insulin clearance (**a**) and hepatic extraction fraction (**b**) by glucose tolerance status during an extended OGTT in 61 individuals with PI-CF. Data are presented as means (95% CI) calculated with unadjusted linear regression models using robust SE. Insulin clearance and hepatic extraction fraction were estimated using a minimal model. Outliers are indicated with red numbers, *p<0.05, **p<0.01

Insulin clearance was higher in individuals with CFRD than in those with NGT, while hepatic extraction fraction was unchanged between the glucose tolerance groups (Fig. 4). Individuals with insulin-treated CFRD had the highest insulin clearance (ESM Fig. 4). In adjusted models, insulin sensitivity, glucose tolerance status, HbA_1c_ and maximum OGTT glucose value were all positively associated with insulin clearance but not with hepatic extraction fraction. Moreover, Φ total and Φ static were negatively associated with insulin clearance, as insulin clearance increased by 0.004 min^−1^ for each unit (10^−9^ min^−1^) decline in Φ total in the adjusted model (Table 2). At the same time, Φ total and Φ static were positively associated with hepatic extraction fraction (ESM Table 1). Fixing the hepatic extraction fraction at 50% in the sensitivity model did not influence the associations with insulin clearance, as the association with glucose tolerance status, HbA_1c_, maximum OGTT glucose value, Φ total and Φ static were unchanged (ESM Table 2). Lastly, age was positively associated with insulin clearance and negatively associated with hepatic extraction fraction in the adjusted models (Table 2 and ESM Table 1). However, this association was not significant in the sensitivity model. Insulin clearance correlated positively with the rough estimate of insulin clearance (*R*=0.64, *p*<0.001) (ESM Fig. 5a), but correlated negatively with hepatic extraction fraction (*R*=−0.7, *p*<0.001) (ESM Fig. 5b).

**Table 2:** Associations between metabolic variables and insulin clearance during an extended OGTT in 61 individuals with PI-CF

Variable	Minimal adjusted model^a^			Multivariate model^b^		
	Slope	95% CI	*p* value	Slope	95% CI	*p* value
Age, years	0.007	0.004, 0.010	<0.001	0.007	0.004, 0.010	<0.001
Female sex	0.005	−0.077, 0.086	0.913	−0.019	−0.081, 0.044	0.555
BMI, kg/m^2^	−0.008	−0.023, 0.007	0.279	−0.009	−0.020, 0.003	0.148
HOMA-IR	−0.039	−0.085, 0.008	0.103	−0.019	−0.060, 0.023	0.374
Matsuda index	0.011	0.004, 0.019	0.003	0.008	0.001, 0.015	0.026
Stumvoll index	−1.529	−2.960, −0.097	0.037	−1.788	−3.483, −0.093	0.039
Insulin sensitivity, 10^−4^ pmol^−1^ l^−1^ min^−1^	0.011	0.002, 0.021	0.023	0.012	0.004, 0.021	0.004
Disposition index, 10^–13^ pmol^–1^ l^–1^ min^–2^	0.000	0.000, 0.000	0.529	0.000	−0.001, 0.000	0.118
Φ total, 10^−9^ min^−1^	−0.005	−0.006, −0.003	<0.001	−0.004	−0.006, −0.002	<0.001
Φ dynamic, 10^−9^	−0.0003	−0.0004, −0.0001	0.001	−0.0001	−0.0003, 0.0000	0.080
Φ static, 10^−9^ min^−1^	−0.005	−0.007, −0.003	<0.001	−0.004	−0.006, −0.003	<0.001
HbA_1c_, mmol/mol	0.008	0.004, 0.011	<0.001	0.006	0.002, 0.010	0.007
Glucose tolerance group	0.098	0.021, 0.176	0.014	0.099	0.001, 0.196	0.047
Maximum glucose value, mmol/l	0.014	0.007, 0.021	<0.001	0.013	0.004, 0.022	0.004
Alkaline phosphatase, U/l^c^	0.000	−0.001, 0.000	0.505	0.000	−0.001, 0.001	0.789
GGT, U/l^c^	0.000	0.000, 0.001	0.158	0.000	0.000, 0.001	0.130
eGFR, ml/min per 1.73 m^2c^	−0.002	−0.006, 0.002	0.371	−0.001	−0.004, 0.002	0.469

Data were calculated in linear regression models with insulin clearance as outcome using robust SE

Insulin clearance was estimated using a minimal model. Each row shows the coefficient from a distinct model with the variable as the independent variable

^a^The minimal adjusted models were adjusted for BMI; glucose tolerance status was included as an ordinal variable

^b^The multivariable models were adjusted for BMI, age, sex and insulin sensitivity; glucose tolerance status was included as an ordinal variable

^c^Most recent assessment prior to the OGTT

In ESM Table 3, we summarise Φ total, Φ dynamic, Φ static, insulin sensitivity and disposition index values estimated in this study and in previous studies in individuals without CF with normal glucose tolerance or type 2 diabetes [25, 26].

## Discussion

We investigated insulin secretion, insulin sensitivity, disposition index and insulin metabolism kinetics in individuals with PI-CF using minimal models. Although insulin secretion peaked incrementally later with worsening glucose tolerance, only those with CFRD had a reduced Φ dynamic and Φ static compared with the other groups. Stepwise reduction in insulin sensitivity accompanied progression from NGT to CFRD and the disposition index declined between NGT and EGI and between IGT and CFRD. Moreover, insulin sensitivity was positively associated with insulin clearance but the CFRD group of participants still had a higher insulin clearance compared with the NGT group and Φ total was inversely associated with insulin clearance. This suggests that insulin deficiency may induce increased insulin clearance in CFRD, while the increased insulin clearance does not seem to explain the reduced insulin sensitivity.

Primary insulin deficiency resulting in hyperglycaemia may lead to a relative reduction in insulin sensitivity, possibly due to hyperglycaemia [27] or due to a delayed and mismatched insulin secretion [28]. Consistently, our study showed that those with delayed and/or lower insulin secretion (i.e. IGT and CFRD) had attenuated insulin sensitivity compared with NGT and this decline could not be explained by differences in inflammation and nutritional status. Some [6, 7] but not all [4] previous metabolic clamp studies in CF have also reported a decline in insulin sensitivity in those with glucose intolerance. This indicates that reduced insulin sensitivity might further impair glucose tolerance in individuals with CF accompanied by reduced pancreatic beta cell function. We also found that neither insulin secretion parameters nor insulin sensitivity were able to distinguish EGI from NGT, while the disposition index differed significantly between the two groups, providing further support for a combinatorial role of insulin secretion and insulin sensitivity in the development of abnormal glucose tolerance in CF.

The present study confirms that the marker of hepatic insulin resistance, HOMA-IR [20], is not associated with glucose intolerance in CF as reported in previous studies [10–12, 29]. This suggests that glucose intolerance is predominantly associated with reduced muscle glucose uptake without affecting hepatic insulin sensitivity in CF as previously indicated [6]. This could also explain the absence of significant correlation between HOMA-IR and the modelled insulin sensitivity. Compared with HOMA-IR and the Matsuda index, the Stumvoll index turned out to have the highest correlation with insulin sensitivity among all the indices, probably because this index is less affected by hepatic insulin sensitivity due to exclusion of the fasting glucose value. Indeed, a difference between the contributions made by hepatic and peripheral insulin sensitivity to estimates of insulin sensitivity may explain some of the discrepancies between previous study conclusions regarding insulin sensitivity in CF.

Insulin clearance, similar to insulin sensitivity, is facilitated by membrane-bound insulin receptors, possibly explaining why insulin sensitivity and insulin clearance have been shown to be positively associated [24]. Insulin clearance is also thought to be a saturable process and it has therefore been proposed that insulin clearance increases when low insulin levels are unable to saturate the clearance pathway [15]. Nevertheless, there seems to be an inverse relationship between hepatic and peripheral insulin clearance [30]. The estimate of insulin clearance in this study cannot distinguish between hepatic and peripheral insulin clearance, although the model enabled the assessment of hepatic extraction fraction [30]. We are therefore unable to identify a compartment responsible for alterations in insulin clearance in this study. Still, our results show that insulin clearance is positively associated with insulin sensitivity and negatively associated with insulin secretion. The positive association with insulin sensitivity is in line with the findings of several studies in individuals without CF [30, 31] and the negative association with insulin secretion is also consistent with observations made across different ethnic populations [15, 16, 30]. On the other hand, a previous study did not detect any differences in insulin clearance in those with glucose intolerance in a young CF population (10–25 years) excluding those receiving insulin treatment [14]. It is possible that the different CF subpopulations might explain the differences in our results and theirs.

This study suggests that rapid insulin disappearance does not explain the reduced insulin sensitivity in CFRD and that the increased insulin clearance in CFRD may be explained by insulin deficiency. Thus, insulin deficiency is also likely to explain the association between hyperglycaemia and increased insulin clearance in CF. Still, the mechanisms causing the altered insulin metabolism kinetics in CFRD are not clear and the increased insulin clearance could be either clinically relevant [32] or without importance. Possibly, the insulin deficiency increases the fraction of insulin that passes through the liver into the systemic circulation [33]. This is supported by our results showing that the hepatic extraction fraction was reduced in those with insulin deficiency, although the hepatic extraction fraction did not vary across groups. Moreover, increased insulin clearance may only affect those with severe insulin deficiency and therefore it does not seem to contribute to the progression in glucose intolerance in CF. Hence, the clinical significance of altered insulin metabolism kinetics among those with CFRD remains uncertain.

A surprising finding in this study was that age was positively associated with insulin clearance and negatively associated with hepatic extraction fraction, as a non-CF study reported the opposite associations [34]. However, a previous study in CF also concluded that insulin clearance was positively associated with age [35]. In CF, older individuals may have much greater reduction in insulin secretion than non-CF counterparts. Therefore, we hypothesised that the apparent effect of age seen in this study was in fact associated with worse insulin deficiency in the older participants. Lastly, alkaline phosphatase, GGT and eGFR were not associated with insulin clearance or hepatic extraction fraction, indicating that insulin metabolism kinetics were not influenced by concomitant liver or kidney disease in CF.

This was a single centre study in Denmark, hence this study benefitted from a relatively genetically homogeneous population with normal BMI who were exposed to similar CF treatment over the years. However, whether the selection of a European cohort prevents the generalisation to other ethnicities is not known. This study was also limited by the number of study participants, which could have resulted in a type II error (e.g. when comparing insulin sensitivity in NGT and EGI). In addition, the study was limited by the lack of mathematical models validated in CF and modelled estimates might therefore deviate from gold standard estimates obtained by metabolic clamps. However, the models have been used in other populations with diabetes and we have no reason to believe that the models are biased when applied to CF. Another limitation was the assumption that hepatic extraction fraction remains constant throughout the OGTT, suggesting that Watanabe’s model provides only relatively crude estimates of insulin clearance and hepatic extraction fraction. Furthermore, age and female sex are associated with glucose intolerance in CF and reduced insulin sensitivity, hence we cannot reject that there may have been some confounding by these factors. Still, there was no significant sex difference across groups, and this suggests that our findings are applicable to both sexes.

In summary, this study confirmed that glucose intolerance in individuals with PI-CF was associated with delayed insulin secretion. Only individuals with CFRD had significantly reduced first-phase and second-phase insulin secretion compared with the other glucose tolerance groups in CF. The study also indicated that peripheral insulin sensitivity and disposition index diminished with worsening glucose intolerance, while the hepatic insulin sensitivity seemed unrelated to glucose tolerance status. Furthermore, our CFRD group appeared to have the highest insulin clearance, possibly due to insulin deficiency, without any association with liver or kidney dysfunction. The insulin deficiency may be the underlining cause of the observed changes in both the peripheral insulin sensitivity and the insulin clearance in CF, although the clinical relevance of these metabolic changes remains unknown.

## Supplementary Information

Below is the link to the electronic supplementary material.

Supplementary file1 (PDF 701 KB)

## Data Availability

The datasets generated during and/or analysed during the current study are not publicly available due to reasons of sensitivity and data protection regulations but are available from the corresponding author upon reasonable request. Data are located in controlled access data storage in REDCap at Region Hovedstaden, Denmark.

## Abbreviations

CF: Cystic fibrosis
CFRD: CF-related diabetes
EGI: Early glucose intolerance
GGT: γ-Glutamyl transferase
IGT: Impaired glucose tolerance
NGT: Normal glucose tolerance
PI-CF: Pancreatic-insufficient CF
