# Supplementary material for: Insulin sensitivity, disposition index and insulin clearance in cystic fibrosis: a cross-sectional study
Source: Diabetologia. 2024 Aug 2;67(10):2188–98. doi: 10.1007/s00125-024-06220-6 (PMC11447061; doi:10.1007/s00125-024-06220-6)
Supplement: Supplementary file 1 — Supplementary file1 (PDF 701 KB) [file 125_2024_6220_MOESM1_ESM.pdf]

ESM Methods

The units for insulin and glucose were adjusted to the equations using the conversion factors 6 pmol/l=1 µU/ml and 1 mmol/l=18.018 mg/dl for insulin and glucose, respectively. The equations for calculating insulin resistance and insulin sensitivity with simple indices are shown below.

HOMA-IR:

$$\frac{\text{Insulin}_{\text{fasting}}\ (\text{pmol/l}) \times \text{Glucose}_{\text{fasting}}(\text{mmol/l})}{6 \times 22.5}$$

Matsuda index:

$$\frac{10{,}000}{\sqrt{}[\left(\text{Glucose}_{\text{fasting}}\left(\frac{\text{mmol}}{\text{l}}\right) \times \text{Insulin}_{\text{fasting}}\left(\frac{\text{pmol}}{\text{l}}\right)\right) \times \left(\text{Glucose}_{\text{mean}}\left(\frac{\text{mmol}}{\text{l}}\right) \times \text{Insulin}_{\text{mean}}\left(\frac{\text{pmol}}{\text{l}}\right)\right) \times \frac{18.018^2}{6^2}]}$$

Stumvoll index:

$$0.156 - 0.0000459 \times \text{Insulin}_{120}(\text{pmol/l}) - 0.000321 * \text{Insulin}_{\text{fasting}}(\text{pmol/l}) - 0.0054 \times \text{Glucose}_{120}(\text{mmol/l})$$

ESM Results

ESM Table 1: Associations between metabolic variables and hepatic extraction fration (%) during an extended OGTT in 61 individuals with PI-CF

Variable	Minimal adjusted model[a]			Multivariate model[b]		
	Slope	95% CI	*p*-value	Slope	95% CI	*p*-value
Age, years	-0.68	(-1.11, -0.25)	**0.002**	-0.68	(-1.11, -0.25)	**0.002**
Female sex	-5.78	(-16.09, 4.53)	0.266	-4.92	(-14.46, 4.62)	0.306
BMI, kg/m^2	0.57	(-1.11, 2.25)	0.499	0.71	(-0.79, 2.21)	0.347
HOMA-IR	-1.83	(-7.45, 3.79)	0.517	-3.79	(-9.32, 1.75)	0.176
Matsuda index	-0.11	(-0.84, 0.62)	0.770	0.25	(-0.46, 0.95)	0.487
Stumvoll index	39.49	(-124.55, 203.53)	0.632	-4.55	(-249.37, 240.29)	0.970
Insulin sensitivity, 10^{-4} pmol^{-1} l^{-1} min^{-1}	-0.39	(-1.70, 0.92)	0.554	-0.33	(-1.76, 1.11)	0.651
Disposition index	0.01	(-0.02, 0.04)	0.438	0.02	(-0.03, 0.07)	0.380
Φ total, 10^{-9} min^{-1}	0.307	(0.11, 0.51)	**0.004**	0.253	(0.05, 0.46)	**0.015**
Φ dynamic, 10^{-9}	0.008	(-0.01, 0.02)	0.349	-0.002	(-0.02, 0.02)	0.833
Φ Static, 10^{-9} min^{-1}	0.35	(0.13, 0.57)	**0.003**	0.30	(0.08, 0.52)	**0.008**
HbA_{1c}, mmol/mol	-0.12	(-0.59, 0.36)	0.627	0.08	(-0.42, 0.59)	0.741
Glucose tolerance group	-2.67	(-12.32, 6.98)	0.582	2.37	(-11.39, 16.13)	0.731
Maximum glucose value, mmol/l	-0.38	(-1.29, 0.52)	0.402	-0.09	(-1.25, 1.07)	0.882
Alkaline phosphatase, U/l[c]	0.01	(-0.08, 0.11)	0.776	0.01	(-0.09, 0.10)	0.917
GGT, U/l[c]	0.00	(-0.05, 0.04)	0.871	0.00	(-0.05, 0.04)	0.844
eGFR, ml/min per 1.73 m^2[c]	0.15	(-0.25, 0.54)	0.460	0.04	(-0.19, 0.28)	0.720

ESM Table 1: Data were calculated in linear regression models with hepatic extraction fraction as outcome using robust SE

Insulin clearance was estimated using a minimal model. Each row shows the coefficient from a distinct model with the variable as the independent variable

[a]The minimal adjusted models were adjusted for BMI; glucose tolerance status was included as an ordinal variable

[b]The multivariable models were adjusted for BMI, age, sex and insulin sensitivity; glucose tolerance status was included as an ordinal variable

[c]Most recent assessment prior to the OGTT

ESM Table 2: Associations between metabolic variables and insulin clearance assuming a fixed hepatic extraction of 50% during an extended OGTT in 61 individuals with PI-CF

Variable	Minimal adjusted model[a]			Multivariate model[b]		
	Slope	95% CI	*p*-value	Slope	95% CI	*p*-value
Age, years	0.003	(-0.001, 0.006)	0.098	0.003	(0.000, 0.006)	0.050
Female sex	-0.03	(-0.08, 0.03)	0.362	-0.05	(-0.12, 0.02)	0.134
BMI, kg/m^2	0.00	(-0.01, 0.01)	0.751	0.00	(-0.01, 0.01)	0.965
HOMA-IR	-0.05	(-0.08, -0.01)	**0.010**	-0.04	(-0.06, -0.01)	**0.009**
Matsuda index	0.01	(0.00, 0.02)	**0.026**	0.01	(0.00, 0.02)	**0.009**
Stumvoll index	-1.54	(-3.08, -0.01)	**0.049**	-2.40	(-3.66, -1.14)	**<0.001**
Insulin sensitivity, 10^{-4} pmol^{-1} l^{-1} min^{-1}	0.01	(0.00, 0.03)	0.103	0.01	(0.00, 0.03)	0.062
Disposition index	0.000	(0.00, 0.00)	0.489	0.000	(0.00, 0.00)	0.055
Φ total, 10^{-9} min^{-1}	-0.003	(-0.005, -0.001)	**0.002**	-0.003	(-0.004, -0.001)	**<0.001**
Φ dynamic, 10^{-9}	-0.0002	(-0.0004, -0.0001)	**0.002**	-0.0002	(-0.0003, 0.0000)	**0.015**
Φ Static, 10^{-9} min^{-1}	-0.003	(-0.005, -0.001)	**0.003**	-0.003	(-0.005, -0.001)	**<0.001**
HbA_{1c}, mmol/mol	0.01	(0.00, 0.01)	**0.003**	0.01	(0.00, 0.01)	**<0.001**
Glucose tolerance group	0.08	(0.01, 0.14)	**0.023**	0.13	(0.04, 0.21)	**0.004**
Maximum glucose value, mmol/l	0.01	(0.00, 0.02)	**0.003**	0.02	(0.01, 0.02)	**<0.001**
Alkaline phosphatase, U/l[c]	0.00	(0.00, 0.00)	0.306	0.00	(0.00, 0.00)	0.966
GGT, U/l[c]	0.00	(0.00, 0.00)	**0.004**	0.00	(0.00, 0.00)	**<0.001**
eGFR, ml/min per 1.73 m^2[c]	0.00	(0.00, 0.00)	0.327	0.00	(0.00, 0.00)	0.242

ESM Table 2: Data were calculated in linear regression models with insulin clearance as outcome using robust SE

Insulin clearance was estimated using a minimal model with fixed hepatic extraction fraction at 50%. Each row shows the coefficient from a distinct model with the variable as the independent variable

[a]The minimal adjusted models were adjusted for BMI; glucose tolerance status was included as an ordinal variable

[b]The multivariable models were adjusted for BMI, age, sex and insulin sensitivity; glucose tolerance status was included as an ordinal variable

[c]Most recent assessment prior to the OGTT

ESM Table 3: Modelled metabolic variables in the traditional units in individuals with and without CF during an extended OGTT

	Non-CF[a]		CF	
	Non-diabetic[1]	Type 2 diabetes[2]	NGT	CFRD
Body mass index (kg/m^2)	26.2±0.6	31.4±4.3	22.5±1.1	22.2±1.1
Φ total (10^{-9} min^{-1})	55±2	6±2	45±4	11±3
Φ dynamic (10^{-9})	872±46	112±21	172±2	11±2
Φ static (10^{-9} min^{-1})	42±2	5±1	41±3	10±3
Insulin sensitivity (10^{-4} dl/kg (μU/ml)$^{-1}$ min^{-1})	15±1	5±2	58±2	21±2
Disposition index (10^{-13} dl/kg (μU/ml)$^{-1}$ min^{-2})	769±66	8±2	1116±167	9±6

ESM Table 3: Data were reported as means ± SE. The volume of distribution was assumed to be 1.43 dl/kg

[a]pmol/l was converted to μU/ml by dividing by 6

[1](Dalla Man, 2005), [2](Geragotou, 2016)

ESM Figure 1:

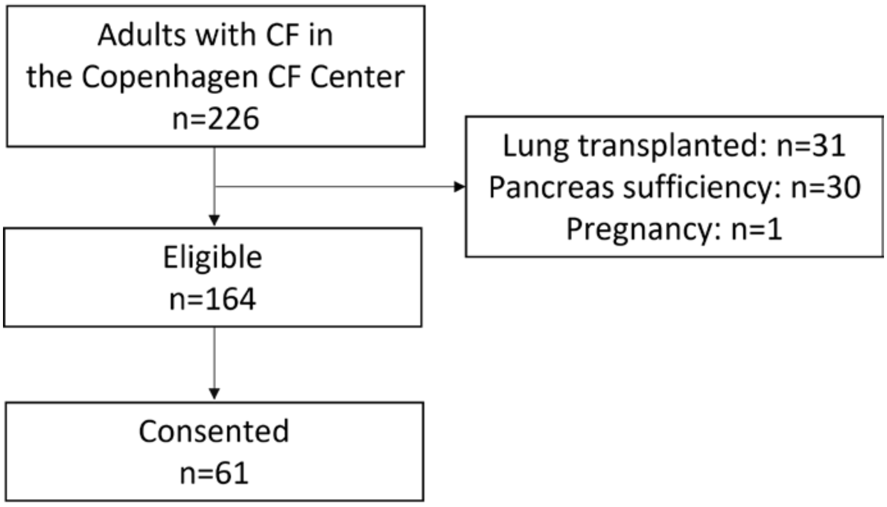

ESM Figure 1: Flow-chart for study participants

ESM Figure 2: Glucose, insulin and C-peptide concentrations by glucose tolerance status during an extended OGTT in 61 individuals with PI-CF

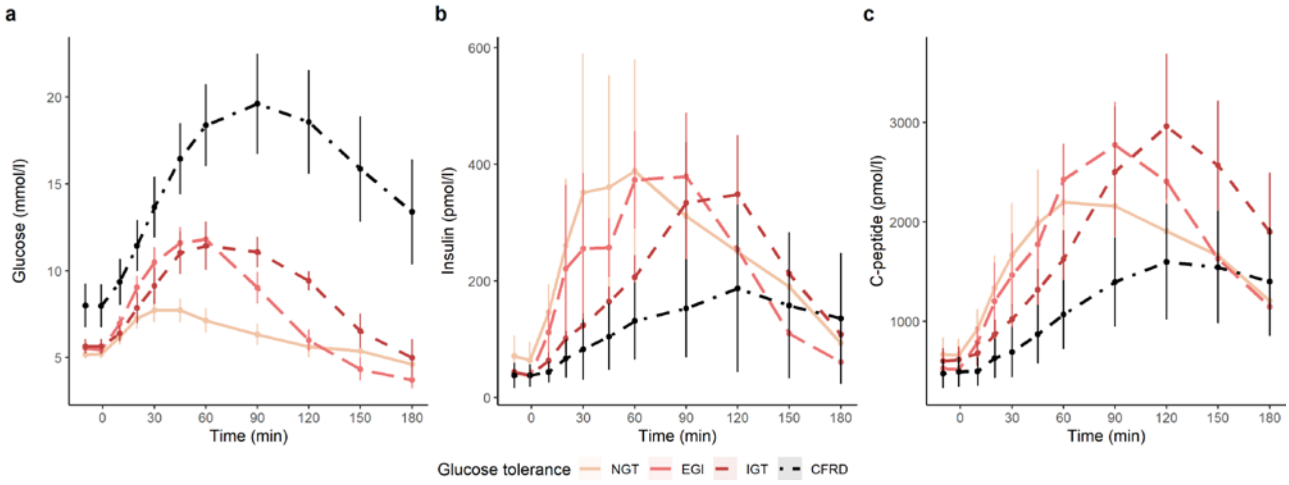

ESM Figure 2: The figures show means (95% CI) of the raw concentrations calculated with unadjusted linear regression models using robust SE

ESM Fig. 3: Time to maximum and the maximum values of the dynamic and static insulin secretion during an extended OGTT in 61 individuals with PI-CF

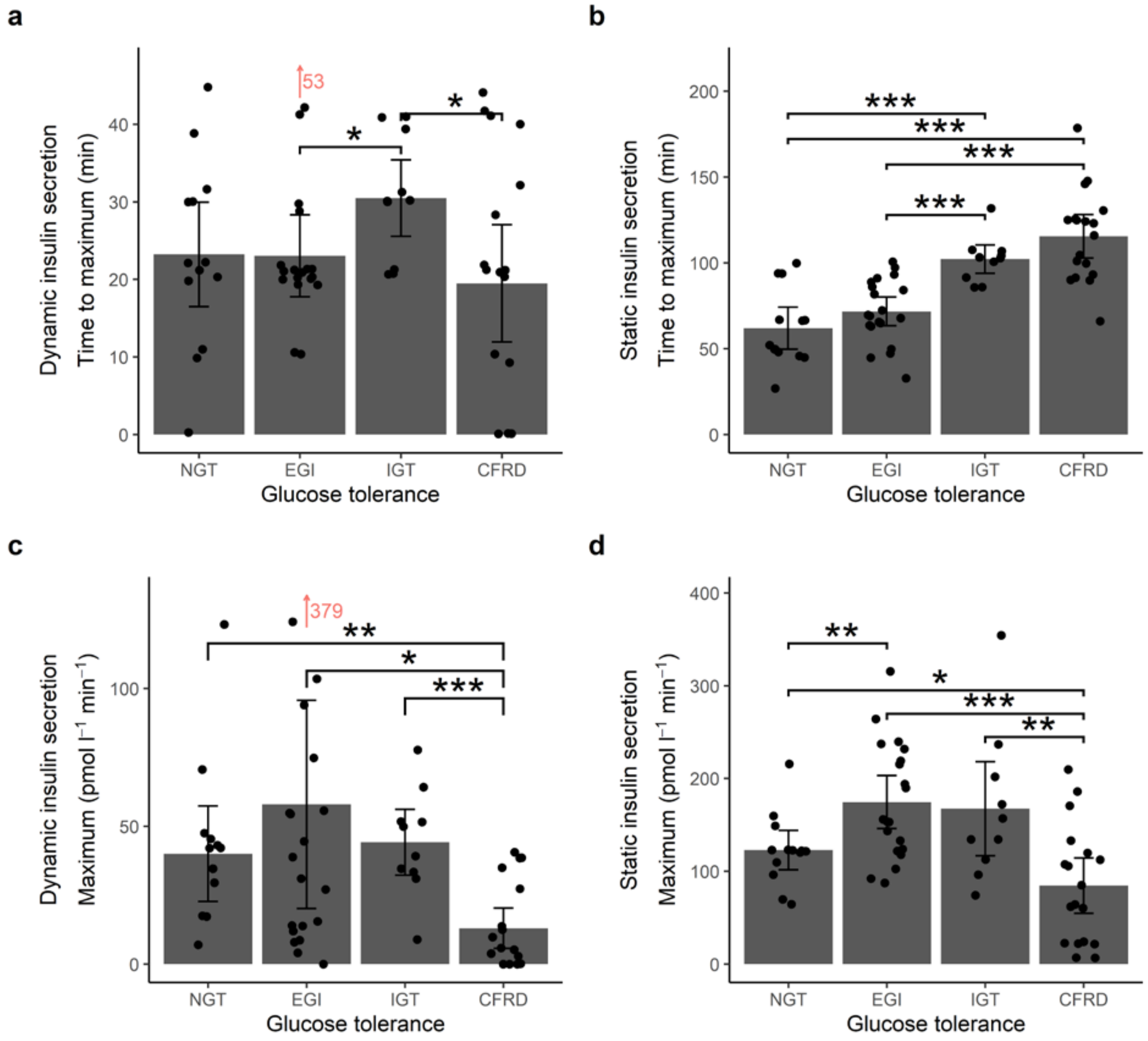

*ESM Fig. 3: The figures show means (95% CI) calculated with unadjusted linear regression models using robust SE. Only significant differences are reported with p-values. Outliers are indicated by red numbers. *p<0.05, **p<0.01, ***p<0.001*

ESM Fig. 4: Total insulin secretion, insulin sensitivity, insulin clearance, and hepatic extraction fraction by glucose tolerance status during an extended OGTT in 61 individuals with PI-CF

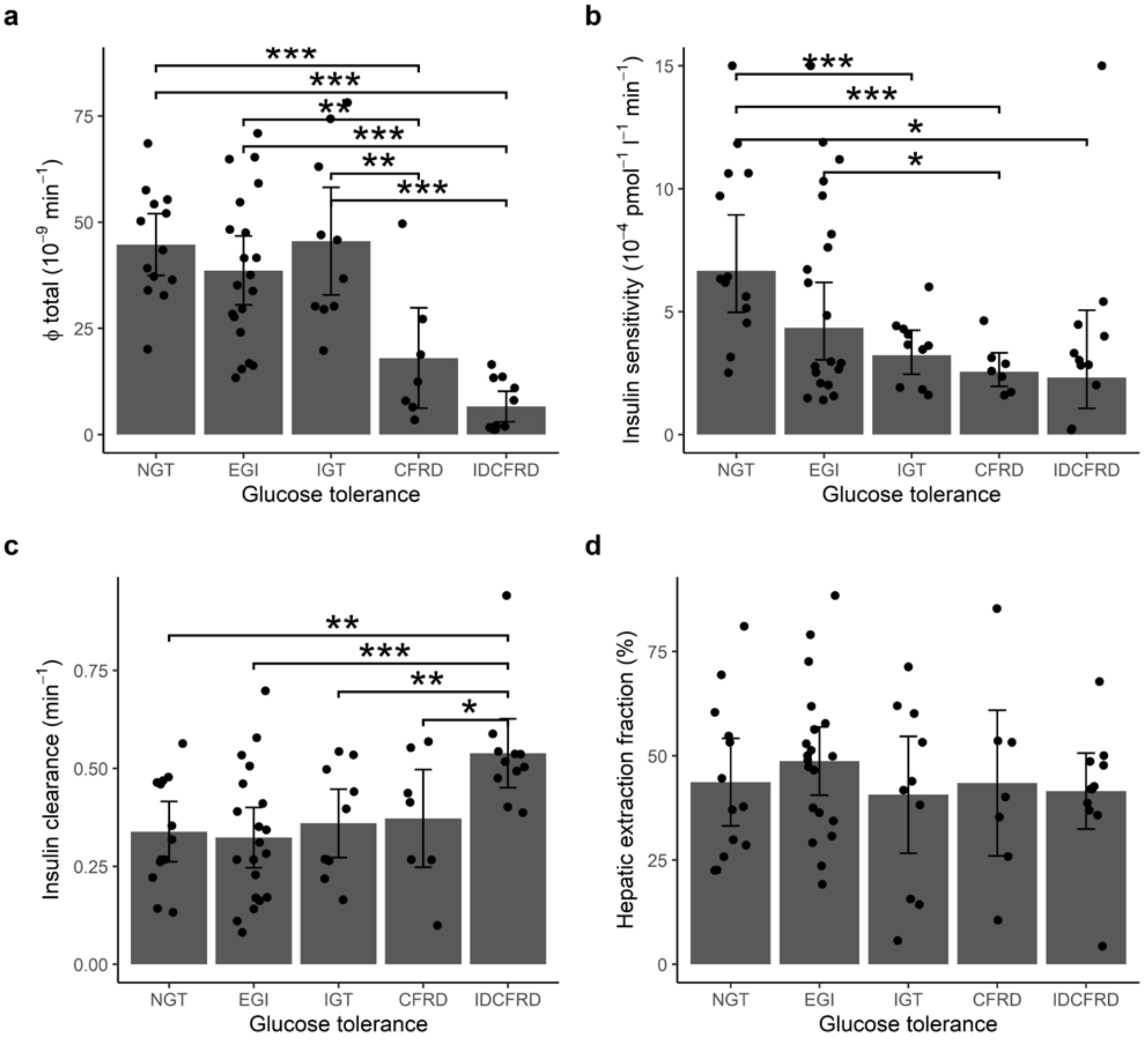

*ESM Fig. 4: Data are presented as means (95% CI) calculated with unadjusted linear regression models using robust SE. Only significant differences are reported with p-values. Insulin sensitivity was log-transformed and back transformed in the model. *p<0.05, **p<0.01, ***p<0.001*

ESM Fig. 5: Spearman's correlation between modelled insulin clearance versus the AUC ratio of C-peptide and insulin and hepatic extraction fraction during an extended OGTT in 61 individuals with PI-CF

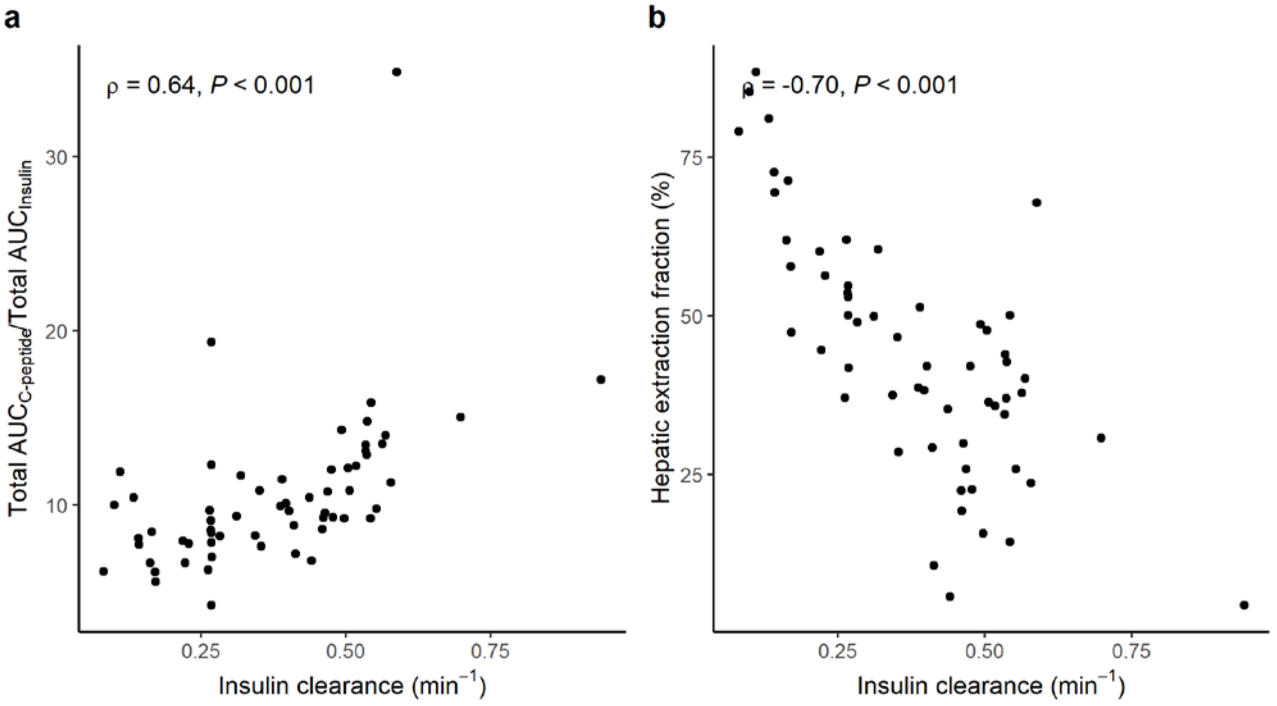

ESM Fig. 5.

References

1. Man CD, Campioni M, Polonsky KS, et al (2005) Two-hour seven-sample oral glucose tolerance test and meal protocol: Minimal model assessment of β-cell responsivity and insulin sensitivity in nondiabetic individuals. Diabetes 54(11):3265–3273. https://doi.org/10.2337/diabetes.54.11.3265

2. Geragotou T, Jainandunsing S, Özcan B, et al (2016) The Relationship of Metabolic Syndrome Traits with Beta-Cell Function and Insulin Sensitivity by Oral Minimal Model Assessment in South Asian and European Families Residing in the Netherlands. J Diabetes Res 2016:1–9. https://doi.org/10.1155/2016/9286303
